# Personalised simulation of hemodynamics in cerebrovascular disease: lessons learned from a study of diagnostic accuracy

**DOI:** 10.3389/fneur.2023.1230402

**Published:** 2023-09-12

**Authors:** Jonas Behland, Vince I. Madai, Orhun U. Aydin, Ela M. Akay, Tabea Kossen, Adam Hilbert, Jan Sobesky, Peter Vajkoczy, Dietmar Frey

**Affiliations:** ^1^Charité Lab for AI in Medicine (CLAIM), Charité-Universitätsmedizin Berlin, Berlin, Germany; ^2^Department of Neurosurgery, Charité-Universitätsmedizin Berlin, Berlin, Germany; ^3^QUEST Center for Responsible Research, Berlin Institute of Health (BIH), Charité-Universitätsmedizin Berlin, Berlin, Germany; ^4^Faculty of Computing, Engineering and the Built Environment, School of Computing and Digital Technology, Birmingham City University, Birmingham, United Kingdom; ^5^Department of Computer Engineering and Microelectronics, Computer Vision and Remote Sensing, Technical University Berlin, Berlin, Germany; ^6^Center for Stroke Research Berlin, Charité-Universitätsmedizin Berlin, Berlin, Germany; ^7^Johanna-Etienne-Hospital, Neuss, Germany

**Keywords:** precision medicine, intracranial atherosclerotic disease, cerebrovascular disease, stroke, cerebral hemodynamics, simulation

## Abstract

Intracranial atherosclerotic disease (ICAD) poses a significant risk of subsequent stroke but current prevention strategies are limited. Mechanistic simulations of brain hemodynamics offer an alternative precision medicine approach by utilising individual patient characteristics. For clinical use, however, current simulation frameworks have insufficient validation. In this study, we performed the first quantitative validation of a simulation-based precision medicine framework to assess cerebral hemodynamics in patients with ICAD against clinical standard perfusion imaging. In a retrospective analysis, we used a 0-dimensional simulation model to detect brain areas that are hemodynamically vulnerable to subsequent stroke. The main outcome measures were sensitivity, specificity, and area under the receiver operating characteristics curve (ROC AUC) of the simulation to identify brain areas vulnerable to subsequent stroke as defined by quantitative measurements of relative mean transit time (relMTT) from dynamic susceptibility contrast MRI (DSC-MRI). In 68 subjects with unilateral stenosis >70% of the internal carotid artery (ICA) or middle cerebral artery (MCA), the sensitivity and specificity of the simulation were 0.65 and 0.67, respectively. The ROC AUC was 0.68. The low-to-moderate accuracy of the simulation may be attributed to assumptions of Newtonian blood flow, rigid vessel walls, and the use of time-of-flight MRI for geometric representation of subject vasculature. Future simulation approaches should focus on integrating additional patient data, increasing accessibility of precision medicine tools to clinicians, addressing disease burden disparities amongst different populations, and quantifying patient benefit. Our results underscore the need for further improvement of mechanistic simulations of brain hemodynamics to foster the translation of the technology to clinical practice.

## 1. Introduction

Intracranial atherosclerotic disease (ICAD) is a highly prevalent chronic condition that ultimately may lead to stroke, a major cause of disability or death worldwide (1). Despite the efforts of large controlled clinical trials, the rate of recurrent stroke from ICAD within 1 year remains as high as 20% for some populations (1–3). Most affected by ICAD are several Asian, Hispanic, and Black communities and because of the ethnic makeup of the world population, the condition may be the most common cause of stroke worldwide (4, 5). It becomes increasingly clear that the prevention of stroke needs to factor in individual characteristics of patients at risk, in contrast to the current prevention paradigm targeting the general population (6). Thus, new methods of precision medicine, an approach tailoring treatment options to individual patient characteristics, are gaining momentum and could help address this healthcare crisis.

Though ICAD is a chronic condition where regular monitoring of disease progression would provide important insights, intracranial disease progression is not typically assessed for numerous reasons. On the one hand, current hemodynamic tests that cover all brain areas are unfit for repeated measurements and come with a list of disadvantages. Bolus-tracking methods such as CT perfusion or dynamic susceptibility contrast (DSC) MRI are hard to standardise, require expert level post-processing, and the administration of potentially harmful contrast agents (7, 8). Arterial spin labelling (ASL) MRI, whilst innovative in nature for using blood as endogenous contrast agent, proved technically challenging and still fails to reach the accuracy needed for broad clinical use (9–11). Currently, the best interventional treatment option is antiplatelet therapy and general risk factor management in secondary prevention of stroke due to ICAD (12–14). These limited options can be attributed to the fact that for developing new therapeutic interventions one must first monitor and identify patients at risk of stroke, especially at moments when interventions are most effective. With the field moving away from the dogma of the grade of stenosis (GoS) as the single most important biomarker for risk stratification in ICAD, the role of picking novel targets for interventions based on individual hemodynamics gains popularity (15). Therefore, novel diagnostic tools for assessing cerebral hemodynamics need to be validated, to ultimately allow for continuous monitoring of disease progression and timely intervention in ICAD.

Precision medicine is an approach where treatments are targeted on the individual characteristics of patients, using genotypic, phenotypic, psychosocial factors or biomarkers (16). Seminal work on precision medicine for cerebrovascular disease has been done by Liebeskind (15) or Rostanski and Marshall (17). The scholars argue that for delivering on the promise of precision medicine in cerebrovascular disease, the plenitude of routinely acquired imaging and clinical data ought to be leveraged. In acute stroke, an image-based assessment of cerebral hemodynamics is already used to identify patients potentially benefitting from thrombectomy therapy well after the established treatment time windows (18). In ICAD, promising image-based methods for hemodynamic assessment are personalised simulations of cerebral blood flow. Using these methods, hemodynamic patterns have emerged that could prove valuable as individual biomarkers of disease progression in ICAD (19–22). These tools utilise structural brain imaging data for mechanistic simulations and allow for an individual assessment of hemodynamic impairment, collateral status, stroke vulnerability and treatment response. One image-based precision medicine framework for cerebrovascular diseases has been proposed by our group previously (22). It comprises four components: (1) patient-specific vessel geometry data from time-of-flight (TOF) MRI, (2) a U-Net deep learning architecture for vessel segmentation, (3) an easy-to-use graphical user interface (GUI) for vessel annotation, all leading to (4) a mechanistic simulation of various parameters of cerebral hemodynamics. Based on an preliminary visual assessment, the framework seemed to provide a promising performance to detect areas vulnerable to ischemia (22). There is, however, a need for quantitative evaluation of mechanistic simulation frameworks to corroborate these findings.

Thus, the aim of this study is to assess the ability of the proposed simulation framework to identify patients with ICAD that are at high risk of subsequent stroke because of their individual cerebral hemodynamics. For this, we measured the accuracy of the simulation in terms of sensitivity, specificity and area under the ROC curve (AUC) to predict areas of hemodynamic vulnerability that were defined by quantitative perfusion imaging with the clinical reference standard DSC-MRI.

## 2. Materials and methods

### 2.1. Study design

We conducted an observational retrospective study to determine the diagnostic accuracy of a previously published simulation framework (22). For this we performed and compared two independent tests of hemodynamic vulnerability for each study subject. The first uses the simulation framework by our group as outlined in 2.4 and 2.5. The second uses DSC-MRI perfusion imaging as reference standard (i.e., ground truth) as described in 2.6. The design of this exploratory validation study is informed by widely accepted guidelines for reporting accuracy studies (23).

### 2.2. Participants

We included patients with ICAD of the PEGASUS trial conducted at Charité-Universitätsmedizin Berlin. Patients have been recruited at the Charité Department of Neurology and affiliated out-patient services between August 2011 and March 2015. Prior to the study all patients gave written informed consent. The study was approved by the authorised institutional review board of Charité-Universitätsmedizin Berlin and was conducted according to the principles expressed in the Declaration of Helsinki. The patient collective has been described before (24–26). Inclusion criteria were (a) unilateral stenosis of the internal carotid artery (ICA) or middle cerebral artery (MCA) of more than 70% according to the European Carotid Surgery Trial (ECST) criteria, (b) age 18–80 years, and (c) clinically and hemodynamically stable status. The grading of all stenoses was confirmed by MR-angiography and/or duplex sonography. For subjects with multiple stenosed vessels only the stenosis with the highest grade ECST was taken into consideration.

### 2.3. Imaging

MR-imaging was performed on a 3 T whole-body system (Magnetom Trio, Siemens Healthcare, Erlangen, Germany) using a 12-channel receive RF coil (Siemens Healthcare, Erlangen, Germany) tailored for head imaging. Subjects who did not undergo all imaging sequences of the protocol had to be excluded from the analysis.

#### 2.3.1. Time-of-flight imaging

The parameters for Time-of-flight (TOF) imaging were: voxel size = 0.5 × 0.5 × 0.7 mm^3^; matrix size: 312 × 384 × 127; TR/TE = 22/3.86 ms; flip angle = 18°; time of acquisition = 3 min 50 s.

#### 2.3.2. Dynamic susceptibility contrast MRI

The Dynamic susceptibility contrast MRI (DSC-MRI) protocol consisted of a series of 80 whole-brain images using a single-shot free induction decay (FID)-EPI sequence. DSC parameters were in detail: field of view = 224 × 224 mm; voxel size = 1.8 × 1.8 × 5 mm^3^; slices = 21; acceleration factor = 2; TR/TE = 1,390 /29 ms; flip angle = 60°; time of acquisition = 1:54 min; 5 mL Gadovist (Gadobutrol, 1 mol/L; Bayer Schering Pharma AG, Berlin, Germany) followed by a 25 mL saline flush, injected using a power injector (Spectris, Medrad, Warrendale PA, United States) at a rate of 5 mL/s. Acquisition parameters are in line with the recommendations by the Acute Stroke Research Imaging Roadmap (27).

#### 2.3.3. Magnetization prepared rapid gradient-echo imaging

As an anatomic reference, a 3D, T1-weighted, magnetization prepared rapid gradient-echo (MPRAGE) sequence with the following parameters was used: voxel size = 1.0 × 1.0 × 1.0 mm^3^; TR/TE = 1,900/2.25 milliseconds; time of acquisition = 4:26 min.

### 2.4. Simulation of brain perfusion

Brain perfusion was simulated *in silico* following the approach previously published by our group (22). TOF images were used as input data representing the individual cerebral vasculature of the study participants. Binary masks were created from TOF images by machine-learning-assisted segmentation with a published U-Net deep learning architecture (28). The segmentations were manually corrected if needed. Skeletons were created as topological representations of the vasculature. The circle of Willis and all major cerebral arteries were annotated up to second branch subsegments using the 3D annotation tool of the simulation framework by our group (22). The annotated skeleton representing the individual cerebral vasculature was used as input to a 0-dimensional simulation model (22). A description of simulation model dimensionality in the context of ICAD can be found below (see Discussion). Users of the simulation are allowed to specify the intracranial pressure (ICP) and systemic blood pressure (BP). Output of the simulation are values of cerebral perfusion pressure $P_{sim}$ in mmHg for the following arterial segments ipsi- and contralateral to the side of stenosis: anterior cerebral artery (ACA) A2 segment, middle cerebral artery (MCA) M2 superior and inferior segments, and posterior cerebral artery (PCA) P2 segment. Only MCA areas were included in this analysis, as the threshold used for the reference test was validated for MCA areas (29).

### 2.5. Areas of vulnerability as defined by the simulation (index test)

**Figure 1:**
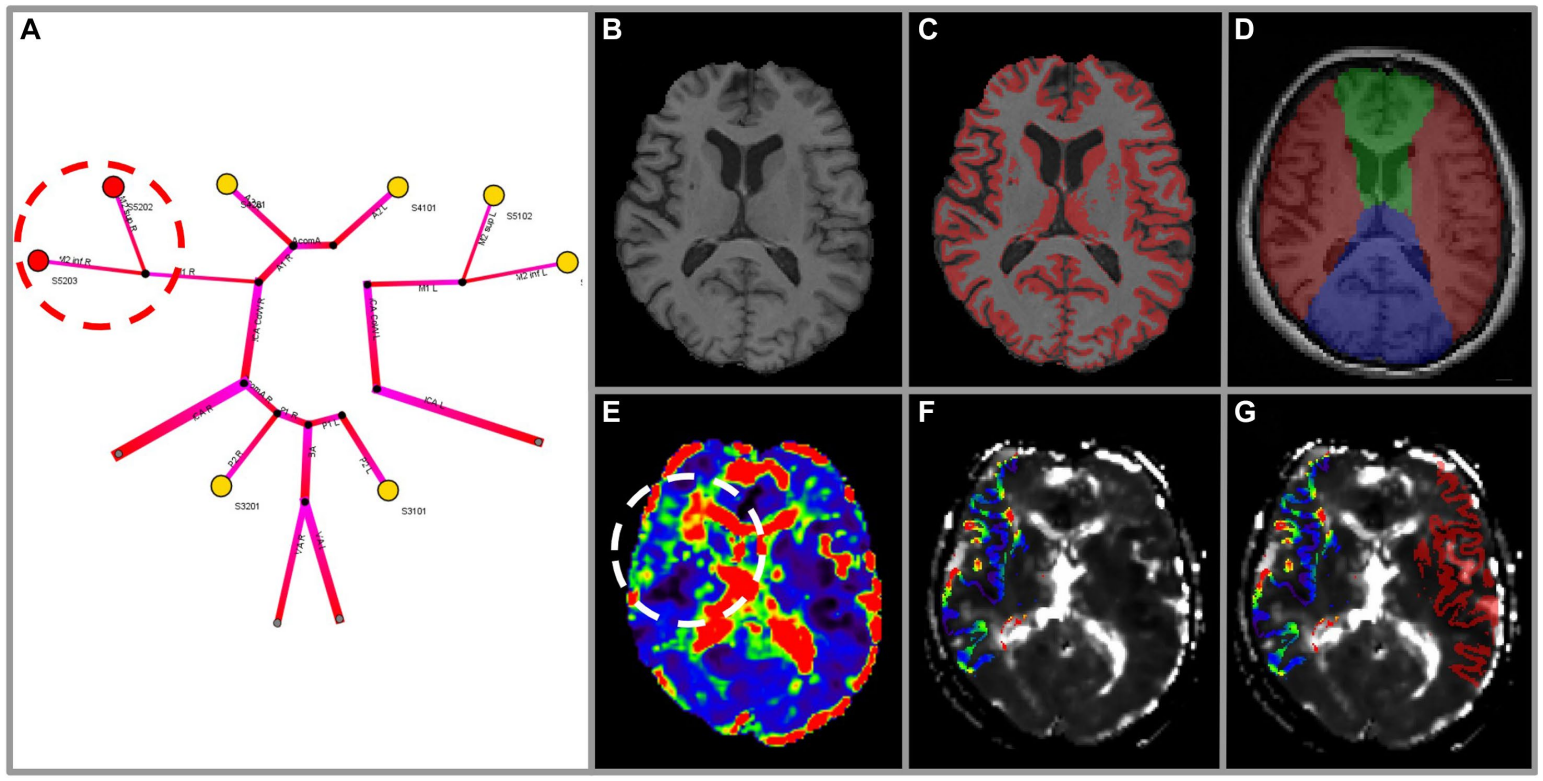
Exemplary methodology of the vulnerability analysis for a subject with high-grade stenosis of the right MCA. Images were highlighted for illustration purposes. The 1-dimensional simulation output **(A)** depicts perfusion pressure $P_{sim}$ for arterial territories (colour-coded yellow ≥ 50 mmHg; red < 50 mmHg). Vulnerability can be seen in the right M2 superior and inferior territories (red circle). Vulnerability according to DSC-imaging is assessed using MPRAGE **(B)** as anatomical reference. Grey matter masking **(C)** and arterial territory masking is performed for ACA, MCA, PCA **(D)** (ACA green, MCA red, PCA blue). Hemispheres are separated in ipsi- and contralateral for normalisation. The mean transit time (MTT) map **(E)** exhibits a clear prolongation in the right MCA territory (white circle). The intersection of masks C and D (right MCA only) is applied to the MTT map **(F)** to denote grey matter of the right MCA territory. Relative values of MTT (relMTT) of the ipsilateral MCA grey matter area are calculated by normalisation with the contralateral MCA grey matter area and then thresholded to denote vulnerability **(G)**.

Vulnerability on both tests of cerebral hemodynamics, the simulation framework and the reference standard, was defined as brain areas unable to compensate for insufficient cerebral perfusion in the event of hemodynamic stroke. The identification of such vulnerable brain areas by the simulation was based on physiological considerations. A mean arterial pressure (MAP) of 70 mmHg was assumed to be the lower limit up to which healthy subjects are able to compensate for hemodynamic impairment keeping regional cerebral perfusion stable (30, 31). This means that in healthy patients an MAP of 70 mmHg is counterbalanced by cerebral autoregulation assuring steady blood supply to the brain. Patients with ICAD, however, are characterised by a reduced capacity to compensate for changes in hemodynamic impairment (32). Thus, we simulated the blood pressure (BP) boundary condition to equal an MAP of 70 mmHg and assessed whether $P_{sim}$ was sufficient in M2 superior and inferior arteries. A segment-level arterial pressure $P_{sim}$ below 50 mmHg in any of these two arteries was used to define vulnerability of the respective MCA segment for this analysis. This corresponds with areas colour-coded red or absent (in case of complete occlusion) on the simulated risk map (see Figure 1A).

### 2.6. Areas of vulnerability as defined by DSC-imaging (reference test)

Resting measurements of mean transit time (MTT), a perfusion parameter, have been shown to be a reliable indicator of hemodynamic impairment in patients with ICAD (33–35). Based on Grubb et al. (29), a side-normalised MTT (relMTT) threshold of 1.387 relative to contralateral healthy tissue of the MCA territory was best at differentiating patients with high risk of subsequent stroke (2-year stroke risk 29.3%) from low-risk patients (2-year stroke risk 4.6%, *p* < 0.001). Hence, a relMTT threshold of 1.387 as measured with DSC-MRI was adopted to denote vulnerability on the reference test of this study. This means that when a patient’s MTT in the MCA area of the side of stenosis was increased by a factor larger than 1.387 compared to the MTT in the respective contralateral MCA area, the reference test was counted as positive (i.e., vulnerable). For this, maps of MTT were created from raw DSC images by parametric deconvolution of a regional concentration time curve with an arterial input function (AIF) (36) using PGui software (version 1.0, Center for functional neuroimaging, Aarhus University, Denmark). For each patient, an AIF was determined by manual selection of three or four intravascular voxels of the MCA M1 segment contralateral to the side of stenosis. AIF shape was visually assessed for peak sharpness, bolus peak time and amplitude width (37, 38). MTT map quality was rated by two independent viewers blinded to clinical data (VIM, senior rater, 10 years of experience in stroke perfusion imaging; JB, junior rater, 1 year of experience). Rating was based on four qualitative criteria: (1) delineation of major cortical structures, (2) presence of susceptibility artefacts, (3) presence of motion artefact, and (4) presence of noise. MTT map quality was rated *very good*–delineation of all structures with no distortions or artefacts; *good*–minimal distortions or artefacts, not affecting the delineation of major cortical structures; *sufficient*–limited presence of distortions or artefacts, partly impairing delineation of major structures, overall preserved diagnostic value; or *insufficient*–extensive presence of distortions or artefacts, prohibiting the delineation of major cortical structures.

Further image processing steps were performed in a Nipype workflow (RRID: SCR_002502): On MPRAGE and DSC images we performed (1) intensity non-uniformity correction (FreeSurfer version 6.0.0, RRID: SCR_001847), (2) skull-stripping (FSL version 6.0, RRID: SCR_002823, fractional intensity threshold = 0.6, manual editing applied in cases of error), and (3) co-registration. Only cortical grey matter and basal ganglia regions were included in this analysis to account for perfusion differences between grey matter and white matter. (4) Grey matter masks were created on MPRAGE using the FAST tool (FSL version 6.0, RRID: SCR_002823; see Figures 1B,C). (5) MCA flow territory masks were created manually according to the atlas of Tatu et al. (39) (see Figure 1D). (6) Both grey matter masks and MCA flow territory masks were applied to MTT maps and the intersection was used to define volumes of interest. (7) Values of relMTT were calculated dividing median MTT ipsilateral to the side of stenosis by median MTT of contralateral healthy tissue. MCA areas with a relMTT above 1.387 were considered vulnerable to subsequent stroke for this analysis (see Figures 1E–G). Performers and readers of both simulation (index test) and DSC-MRI (reference test) were blinded to clinical information. The Python code for all image processing steps is available on Github.^1^

### 2.7. Statistical analysis

The above presented analysis leads to a binary assessment of yes/no for the existence of vulnerability for each patient by the simulation and the reference test. Performance of the simulation to detect vulnerability was assessed by calculating sensitivity and specificity to detect vulnerabilities as defined by DSC-MRI. To account for class imbalances measures of F1-score and geometric mean of sensitivity and specificity were calculated. 95% confidence intervals for sensitivity and specificity were calculated. ROC curves of varying $P_{sim}$ thresholds were created and used to measure the area under the ROC curve (AUC). The optimal threshold for simulation vulnerability was chosen as the point on the ROC curve giving the highest geometric mean of sensitivity and specificity. All statistical analyses have been conducted in Python using the scikit learn library (RRID: SCR_002577). Plots of ROC curves were created using the matplotlib library (RRID: SCR_008624). The Python code of the statistical analysis is available on the Github repository provided above. A diagram showing the flow of participants was created in Lucidchart.^2^ Subjects with missing on either index or reference test were excluded from the analysis. For this exploratory retrospective analysis there was no *a priori* estimate of effect size.

## 3. Results

### 3.1. Participants

**Figure 2:**
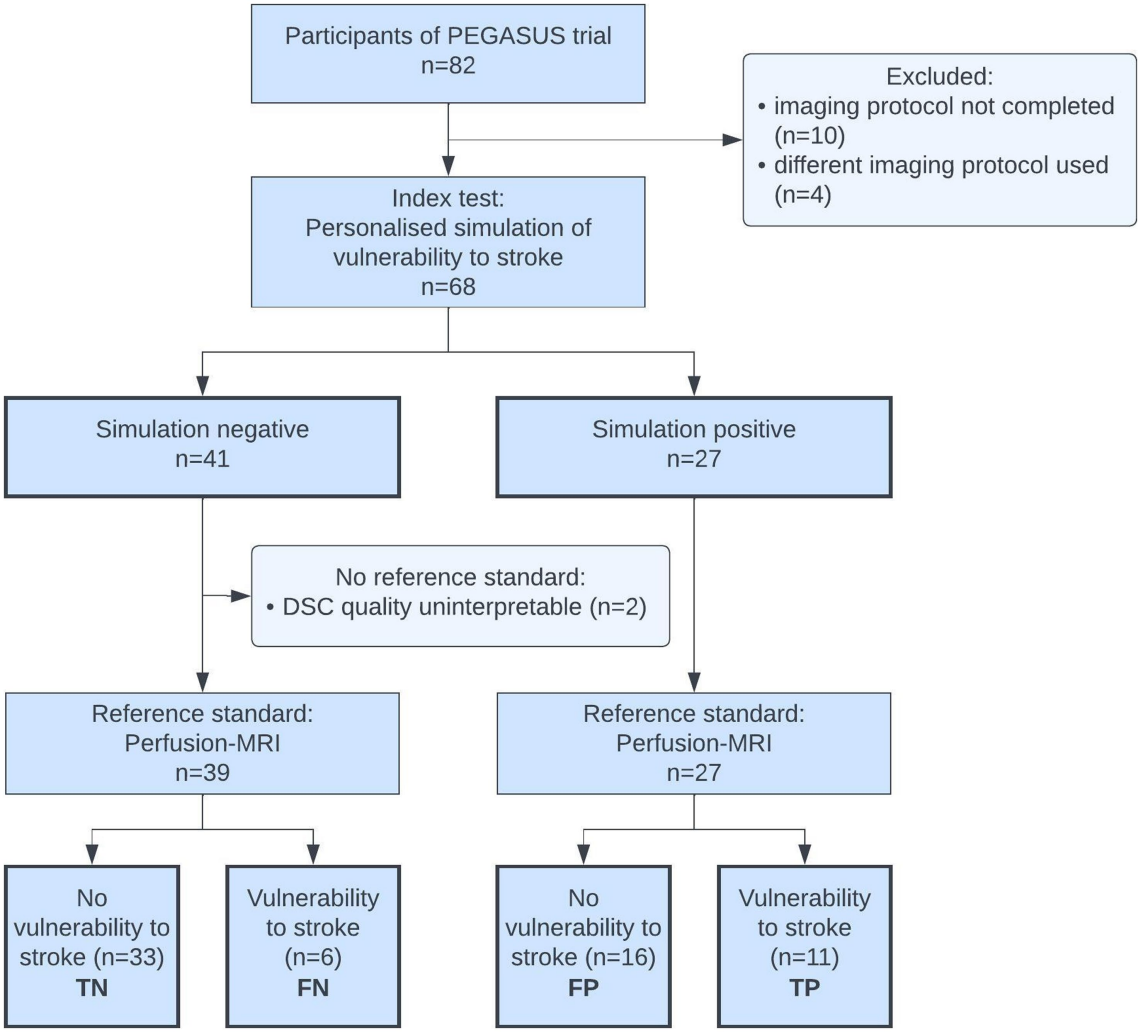
Flow of participants diagram. TN, true negatives; FN, false negatives; FP, false positives; TP, true positives.

We were able to include 68 of 82 cases with ICAD from the PEGASUS cohort. Exclusion were made only when missing or different imaging data did not allow for the creation of the simulation of vulnerability or creation of the reference test. A total of 14 cases had to be excluded for reasons of missing or incomplete DSC or TOF-MRI (10 cases), or a difference in TOF-MRI protocol (4 cases). Vulnerability to stroke status was successfully simulated for all 68 subjects following the precision medicine framework of our group (22). DSC-MRI could be used as a reference test in 66 of the 68 cases. In 2 of the 68 cases, we could not perform the reference test as the DSC-MRI parameter map quality was insufficient for analysis. A detailed quality analysis of the reference standard is provided below. The flow of participants can be seen in Figure 2.

Of all subjects 39.7% identified as female, median age was 57 years (range 29–82 years). Two thirds of the subjects suffered from stroke prior to the study, irrespective of aetiology; 17.6% had a prior transient ischemic attack (TIA). Summary statistics for demographic and clinical characteristics are shown in Table 1. Left ICA was the vessel with the highest grade stenosis (GoS) in 53% of all subjects (36 cases), followed by right ICA in 31% (21 cases), left MCA in 10% (7 cases), and right MCA in 6% (4 cases), respectively. The average GoS amongst these was 89% according to ECST. Clinical data for all subjects is provided in Supplementary Table 1.

**Table 1:** Summary of baseline demographic and clinical characteristics of participants in the PEGASUS trial.

Patient characteristics	*n* (%)
Subjects of PEGASUS trial	
Total	82
Included	68
Median age, years (range)	57 (29–82)
Women	27 (39.7)
Mean NIHSS, points (range)	1.1 (0–11)
Mean mRS, points (range)	0.6 (0–4)
Mean Barthel index, points (range)	97.6 (40–100)
Prior vascular event	
Stroke	46 (67.6)
Transient ischemic attack	12 (17.6)
Side of highest grade stenosis	
Left	40 (58.8)
Right	28 (41.2)

NIHSS, National Institutes of Health Stroke Scale; mRS, Modified Rankin Scale.

### 3.2. Quality of reference standard

DSC parameter map quality of 68 cases was visually assessed by two independent raters; consensus rating was reached in all cases. 97% of DSC MTT maps were rated either very good (25%), good (44%), or sufficient (28%) and thus included for analysis. However, 22 of the 68 cases (32%) showed motion artefacts on visual analysis, diminishing reference standard quality. In six of the 68 cases (9%) the motion artefact exhibited a clear left-to-right heterogeneity, skewing side-wise measures of relMTT. In 2 of 68 cases (3%) DSC quality was rated uninterpretable due to low signal-to-noise ratio, resulting in no reference standard for these subjects.

### 3.3. Test results

On DSC-MRI 17 of 66 cases exhibited vulnerability to stroke with elevated relMTT above 1.387. In the simulation we detected 11 of these 17 cases of vulnerability, representing a true positive rate (TPR) of 64.7% and false negative rate (FNR) of 35.3%. 49 cases exhibited relMTT of less than 1.387 on DSC-MRI and were thus not vulnerable to stroke. 33 of these 49 cases were correctly identified by the simulation as not vulnerable, corresponding to a true negative rate (TNR) of 67.4% and a false positive rate (FPR) of 32.7% (see Table 2).

**Table 2:** Confusion matrix of the simulation results following the framework by our group (22) with the results of the reference test.

Simulation (index test)	Vulnerability to stroke by DSC-MRI (reference test)		Total
	Positive	Negative
Positive	11	16	27
Negative	6	33	39
Total	17	49	66

**Figure 3:**
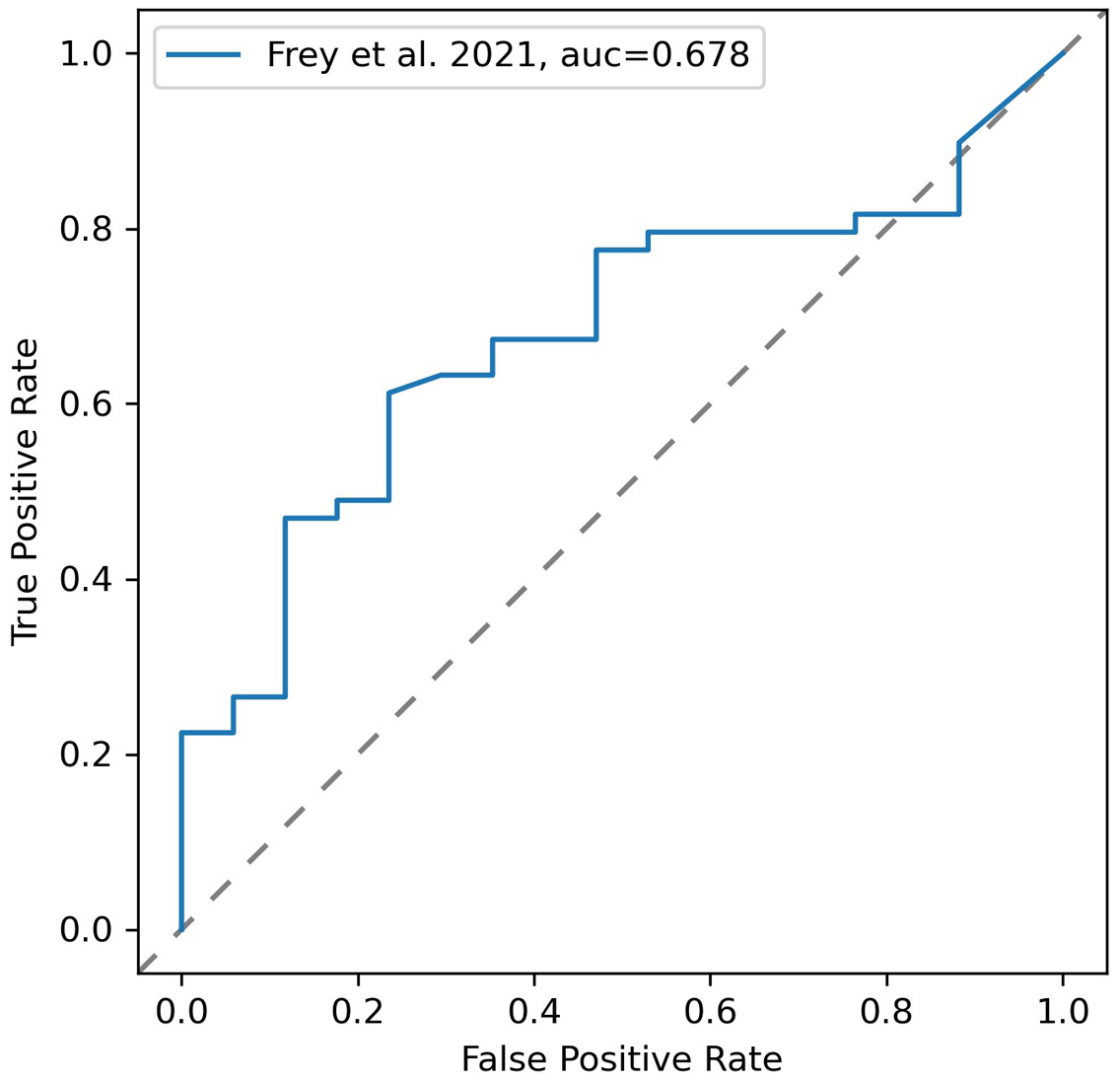
Receiver operating characteristic (ROC) curve for varying thresholds of simulated vulnerability using areas defined as vulnerable by DSC-MRI as reference test.

Based on these results, the sensitivity of the simulation to detect patients vulnerable to stroke in MCA-territory as denoted by DSC perfusion-MRI was 0.647, 95% CI [0.420, 0.874]. The specificity was 0.674, 95% CI [0.542, 0.805]. The overall accuracy of the simulation was 0.667. The geometric mean of sensitivity and specificity was 0.66 with an F1-score of 0.5. A ROC curve for varying thresholds of $P_{sim}$ is shown in Figure 3. The ROC-AUC of the simulation was 0.678. The optimal $P_{sim}$ threshold to denote vulnerability on the simulation was 50.48 mmHg at a geometric mean of sensitivity and specificity of 0.684. The optimal $P_{sim}$ threshold varied just marginally from the physiologically assumed threshold of 50 mmHg.

### 3.4. Distribution of severity of disease in those with vulnerability to stroke

Of the 17 patients with vulnerability to stroke on DSC-MRI, 10 showed full occlusion of the examined vessel (59%), 13 suffered from prior stroke (77%), one from prior TIA (6%). Of the 51 patients without vulnerability to stroke on DSC-MRI, 26 showed full occlusion (51%), 33 suffered from prior stroke (65%), 11 from prior TIA (22%). No information on alternative diagnoses of participants was available.

### 3.5. Time interval and any clinical interventions between index test and reference standard

Imaging for both simulation (TOF, MPRAGE) and reference test (DSC, MPRAGE) has been acquired in one session. No clinical interventions other than the ones needed for imaging, i.e., administration of contrast agent, have been performed in between index test and reference standard.

## 4. Discussion

We performed the first quantitative validation of a framework for the personalised simulation of cerebral hemodynamics in patients with ICAD against a clinical perfusion imaging standard. The presented results indicate a low-to-moderate performance of the simulation framework to predict hemodynamic vulnerabilities. Whilst simulation approaches for hemodynamic assessment of patients with chronic cerebrovascular disease remain promising, our results indicate that further research is required to improve their performance.

ICAD is a complex disease, potentially progressing to stroke through various separate mechanisms. Strokes in ICAD can occur as *in situ* thrombotic occlusion, artery-to-artery embolism, hemodynamic insufficiency or branch occlusion (40). Individualised mechanistic simulations of cerebral perfusion pay tribute to this fact by establishing biomarkers of cerebral hemodynamics which can be used to individualise treatment and secondary stroke prevention. They can both deepen our understanding of the hemodynamic properties of different subtypes of ICAD and allow for scenario analyses, evaluating individual effects of interventions. In the following we would like to elaborate on a number of potential use cases for personalised hemodynamic simulations. Firstly, individual BP management in ICAD and other chronic cerebrovascular diseases. BP control is a central but highly debated element of secondary stroke prevention in patients with symptomatic ICAD (sICAD). The latest guidelines for secondary prevention of stroke favour a target systolic BP < 140 mmHg, whilst noting that most sICAD patients would benefit from even lower BP levels (10, 41). There is, however, a subgroup of sICAD patients with established hemodynamic impairment that, when administered to an antihypertensive regime targeting < 140/90 mmHg, exhibit an increased risk of ipsilateral stroke recurrence (42, 43). If improved, precision medicine tools as the one assessed in this study may be used for establishing individual BP management strategies in ICAD. Secondly, patient selection for interventional trials. Endovascular therapy for ICAD using angioplasty with and without stenting is currently reserved for individual cases of sICAS with multiple recurring strokes despite aggressive medical treatment. It is worth noting that several studies failed to establish a benefit of endovascular therapy compared with best medical treatment, when selecting patients on the basis of grade of stenosis or clinical data (i.e., recent strokes due to ICAD) (3, 13, 14). Here, hemodynamic simulations could act as a perfusion-based biomarker better capturing individual patterns of clinically relevant hemodynamic impairment in ICAD. This could be used for patient selection in trials of promising new interventional therapies for ICAD, whether endovascular such as submaximal angioplasty (44), surgical such as encephaloduroarteriosynangiosis (EDAS) (45), or other. Thirdly, simulation of post-interventional status. In cases of carotid occlusion or moyamoya disease, where lumen-opening procedures or bypasses are used more frequently, mechanistic simulations could help anticipate the post-interventional effects on an individual patient’s cerebral hemodynamics. A feature for this has been included in the simulation framework under investigation but requires further clinical validation (22). Lastly, early detection of ICAD. Precision medicine tools based on non-harmful imaging methods may allow for frequent monitoring and early detection of asymptomatic ICAD, helping to prevent the often unnoticed cognitive decline due to silent infarctions that typically precede disabling ischemic events (1).

Different simulation models for cerebral hemodynamics have been proposed in the literature, which can be categorised based on the dimensionality of the modelling approach. 3D models, also referred to as computational fluid dynamics (CFD) models, were shown to be a powerful tool for numerical representation of complex flow patterns in single cerebral arteries or lesions in ICAD (20, 46–48), intracranial aneurysms (49), or moyamoya disease (50). These studies identified flow abnormalities such as increased wall shear stress, elevated plaque steepness or low fractional flow as indicators of hemodynamic impairment. However, CFD models require vast computational resources, narrowly defined boundary conditions, and expert supervision, currently making them research-oriented solutions with limited applicability for broad use in the clinical setting. 1D or 0D models (i.e., reduced order models) can help overcome these issues by reducing the complexity of simulations. 1D models simulate pressure and velocity changes along a single spatial dimension (i.e., the length of a vessel) and thus allow for the analysis of pulse wave transmission inside the vasculature, requiring less computation resources. 1D models were presented simulating the effects of occlusions or anatomical variations on the circle of Willis hemodynamics (51, 52). 0D models, or lumped models, break the vascular system into functional objects (i.e., organs, vessels) connected by resistances, inductances, and capacitances—analogously to electrical circuits. They allow for rapid simulations of large systems like whole-brain hemodynamics, using only a fraction of the resources of higher-dimensional models. The framework proposed by our group follows this approach by enriching a 0D lumped model with subject-specific vessel geometry data from TOF-MRI (i.e., vessel length and radius). By doing so, the framework allows for patient-specific modelling and achieves fast runtimes using little computing resources, thus making it feasible for use on off-the-shelf computers and mobile devices. Unlike other models, the framework omits personal boundary conditions (i.e., inlet flow, vascular resistance) in favour of user-defined values for BP and intracranial pressure (ICP) (21). Thereby users can simulate scenarios of interest such as global normotension, critical hypotension or increased ICP.

Based on such theoretical considerations, lumped models may mark a significant leap towards a personalised medicine approach in care for patients with cerebrovascular diseases. Our empirical results, however, indicate that the innovative simulation approach under investigation is still exploratory and needs further refinement to change clinical practice. Based on the data presented, the accuracy for personalised simulation of cerebral hemodynamics did not reach the clinical reference standard DSC-MRI. A low-to-moderate sensitivity and specificity imply that the framework is not yet able to detect patients at high risk of subsequent stroke reliably. This may be attributed to the fact that mechanistic simulations of highly complex systems make assumptions for reasons of computational feasibility. Firstly, blood flow is assumed to be Newtonian. However, the rheological properties of blood, a non-Newtonian fluid, have been shown to influence local hemodynamics at vessel bifurcations and narrowings (53). This has important implications on wall shear stress, a governing driver of disease progression in ICAD that is currently not addressed by the simulation. Secondly, the simulation assumes vessel walls to be rigid tubular structures. In reality, vessels can be highly irregular and compliant to changes in pressure and states of brain activity (54). In ICAD, arteries are capable of remodelling in reaction to plaque formation, further increasing complexity in flow patterns (55). Thirdly, TOF-MRI is assumed to be a perfect representation of a subject’s vasculature. TOF-MRI is well-known to suffer from partial signal loss on stenotic vessels due to spin dephasing and partial volume effects, limiting the measure of vessel diameter (56–58). These measures of vessel diameter, however, are central to the simulation framework, where even small inaccuracies in diameter may lead to profound changes in $P_{sim}$ as the pressure inside a vessel is inversely proportional to the fourth power of its radius, following Hagen–Poiseuille equations. Lastly, cerebral blood flow simulations have been shown to be highly sensitive to narrowly defined boundary conditions of inflow and outflow (59–61). As a boundary condition the simulation framework by our group takes a user-specified MAP. Whilst this allows for scenario analysis with varying MAP, it does not necessarily match the individual conditions present at the time of image acquisition.

What could now be the way forward for simulation methods in ICAD? There are various research opportunities on how to close the gap to clinical practice. Firstly, to increase diagnostic power researchers may consider incorporating additional patient information into their applications. Future work could explore the use of readily available multi-modal data sources, both clinical, imaging, and administrative. Instead of using transcranial Doppler sonography (TCD) only for determining GoS, as was done in the PEGASUS study, the use of flow measures from TCD as personalised boundary conditions could improve accuracy of our group’s simulation. Shen et al. successfully implemented personalised boundary conditions from TCD flow data for simulating cerebral blood flow (21). Secondly, precision medicine tools for simulation of hemodynamics need to be more accessible to clinicians. This could be done by further automatization of time-consuming user interactions (i.e., vessel segmentation and annotation) or reduced computing burden of simulations. Our group’s framework offers a simple GUI and can be run on off-the-shelf computers, both essential for integration into existing hospital IT-infrastructure. Nevertheless, a precision medicine tool for ICAD with high usability across multiple use cases has yet to be established. Thirdly, precision medicine tools for ICAD should take into account the uneven distribution of disease burden amongst different populations. For this, the cohorts used for calibration and validation have to match the characteristics of patients most affected by ICAD. In the end, it remains to be shown how precision medicine tools in ICAD improve care for Asian, Hispanic, and Black communities. Fourthly, patient benefit needs to be clearly quantified. Outcome measures for this may be clinical (survival, disease control, complications) or patient-reported (quality of life, social participation, mobility). A set of patient-centred outcome measures for stroke has been published by Salinas et al. and could be used for this purpose (62). Fifthly, clinical evaluation with DSC-MRI can be a valuable tool for highlighting areas of improvement and deducing recommendations for future simulation approaches. However, authority approval will eventually require gold-standard validation with ^15^O-water PET or intraoperative flow measurements. Whilst these are expensive and data scarce, artefact-ridden clinical reference standards such as DSC-MRI may pose insufficient as a benchmark for widespread clinical use. Lastly, for patients this currently means that the diagnosis of ICAD continues to rely on potentially harmful perfusion imaging, duplex sonography, and digital subtraction angiography. Thus, there remains an unmet need for safe diagnosis and tracking of disease progression in ICAD.

The results of this study have to be viewed in light of several limitations. First, the accuracy of the used DSC-MRI reference standard has to be considered inferior to ^15^O-water PET (63). Nevertheless, resting measurements of MTT have been shown to be indicative of cerebral hemodynamic impairment in ICAD by various scholars (33–35). Taking PET data from the St. Louis Carotid Occlusion Study, Grubb et al. (29) reported a threshold MTT ratio of 1.387 to best identify subjects with ICAD at risk for subsequent ipsilateral stroke. As the MTT ratio is a relative measure, setting local MTT in relation to contralateral healthy brain tissue, it can be assumed to hold validity in application to MR-based perfusion imaging. The underlying linear relationship between PET-derived and DSC-derived measures of MTT have been shown to hold true for ICAD (64), moyamoya disease (65), and acute stroke (66). Thus, by using a relMTT threshold validated on ^15^O-water PET, this study assures alignment with gold standard perfusion imaging. Second, no motion correction was applied to raw DSC images. As shown, the used DSC-MRI data exhibited substantial motion artefacts in 32% of subjects potentially reducing the accuracy of the simulation. To account for motion-related outlier values of cerebral perfusion on DSC, only median MTT values were included in this analysis. Third, development and validation of the simulation framework largely relied on the same PEGASUS dataset, possibly resulting in overfitting the model to the used training data. Hence, further improvements to the simulation should be evaluated on different data. Lastly, the PEGASUS study did not include information on patient ethnicity. As enrolment took place in Berlin, Germany only, it appears likely that mostly Caucasian subjects were enrolled, raising the question of external validity.

## 5. Conclusion

We performed the first quantitative validation of a lumped simulation model to identify brain areas vulnerable to stroke against the clinical reference imaging standard DSC-MRI. The framework achieved low-to-moderate accuracy to identify brain areas vulnerable to subsequent stroke. Thus, further refinement of this methodology is needed urgently for translation into clinical practice.

## Data availability statement

The data analysed in this study is subject to the following licences/restrictions: data protection laws prohibit sharing of the PEGASUS dataset. Requests to access these datasets should be directed to the Ethical Review Committee of Charité-Universitätsmedizin Berlin, ethikkommission@charite.de.

## Ethics statement

The studies involving humans were approved by Authorised Institutional Review Board of Charité-Universitätsmedizin Berlin. The studies were conducted in accordance with the local legislation and institutional requirements. The participants provided their written informed consent to participate in this study.

## Author contributions

JB, VM, and DF: conceptualization and project administration. JS, PV, and DF: funding acquisition. JB, OA, and EA: data curation. JB, VM, TK, AH, JS, and DF: methodology. JB and OA: programming the Nipype pipeline. JB and VM: writing—original draft. JB, VM, OA, EA, TK, AH, JS, PV, and DF: writing—review and editing. All authors contributed to the article and approved the submitted version.

## Funding

This study has received funding from the European Commission through a Horizon2020 grant (PRECISE4Q grant no. 777 107, coordinator: DF) and the German Federal Ministry of Education and Research through a Go-Bio grant (PREDICTioN2020 grant no. 031B0154 lead: DF).

## Conflict of interest

VM, TK, and AH reported receiving personal fees from ai4medicine outside the submitted work. Whilst not related to this work, JS reports receipt of speakers’ honoraria from Pfizer, Boehringer Ingelheim, Daiichi Sankyo, Gore, Maquet, Paion, Sanofi, Takeda pharma, UCB, and Berlin Chemie. DF reported receiving grants from the European Commission, reported receiving personal fees from and holding an equity interest in ai4medicine outside the submitted work.

The remaining authors declare that the research was conducted in the absence of any commercial or financial relationships that could be construed as a potential conflict of interest.

## Publisher’s note

All claims expressed in this article are solely those of the authors and do not necessarily represent those of their affiliated organizations, or those of the publisher, the editors and the reviewers. Any product that may be evaluated in this article, or claim that may be made by its manufacturer, is not guaranteed or endorsed by the publisher.

## Footnotes

^1^The Github repository can be accessed via https://github.com/prediction2020/icad_simulation_vs_perfusion.

^2^Lucidchart can be accessed online via www.lucidchart.com.
