# Supplementary material for: Personalised simulation of hemodynamics in cerebrovascular disease: lessons learned from a study of diagnostic accuracy
Source: Front Neurol. 2023 Sep 12;14:1230402. doi: 10.3389/fneur.2023.1230402 (PMC10523575; doi:10.3389/fneur.2023.1230402)
Supplement: Supplementary file 1 [file Table_1.pdf]

Supplementary Material

Personalised simulation of hemodynamics in cerebrovascular disease: Lessons learned from a study of diagnostic accuracy

Jonas Behland, Vince I. Madai, Orhun U. Aydin, Ela M. Akay, Tabea Kossen, Adam Hilbert, Jan Sobesky, Peter Vajkoczy, Dietmar Frey*

*** Correspondence:** Dietmar Frey, dietmar.frey@charite.de

1 Supplementary Tables

Supplementary Table 1. Detailed demographic and clinical characteristics of the study subjects at baseline. The data presented here has in part been published by Martin et al. (1) (patient numbers changed). f: female, m: male, y: years, mRS: Modified Rankin Scale, p: points, NIHSS: National Institutes of Health Stroke Scale, TIA: transient ischemic attack, GoS: grade of stenosis according to the European Carotid Surgery Trial (ECST), ICA: internal carotid artery, MCA: middle cerebral artery.

Patient number	Sex	Age (y)	mRS (p)	NIHSS (p)	Stroke	TIA	GoS (%)			
							ICA right	ICA left	MCA right	MCA left
1	m	73	4	2	x		90%	100%		
2	f	70	0	0		x		80%		
3	f	79	2	3		x		70%		
4	f	61	0	0		x	100%	50%		
5	m	45	0	0	x			100%		
6	m	50	0	0		x				100%
7	m	67	0	0	x		70%	100%		
8	f	56	0	0	x			100%		
9	m	42	1	1		x			100%	
10	f	50	0	0			100%			
11	m	59	1	2	x		90%			
12	f	57	0	0	x			100%		

Patient number	Sex	Age (y)	mRS (p)	NIHSS (p)	Stroke	TIA	GoS (%)			
							ICA right	ICA left	MCA right	MCA left
13	f	45	0	0			70%			
14	m	61	1	0	x		60%	100%		
15	m	63	2	4	x		90%			
16	f	49	0	0	x		90%			
17	f	36	4	11	x			100%		
18	m	76	0	0	x		> 70%			
19	m	31	0	0	x			100%		
20	m	78	0	0		x	40-50%	70%		
21	m	47	4	8	x		100%			
22	f	50	0	0	x				80%	
23	f	63	0	0	x		40%	40%	> 70%	50%
24	f	37	0	0				100%		
25	f	49	0	0	x			100%		
26	f	73	0	0			70%	30%		
27	m	48	0	0				70-80%		
28	m	60	0	0			100%	60%		
29	m	46	0	0	x			100%		
30	f	73	0	0		x		>70%		
31	m	60	0	0		x		70-80%		
32	f	29	0	0	x		20-40%	20-40%	70%	80%
33	m	65	0	0				100%		
34	f	74	0	0			80%			
35	m	58	0	0		x		86-99%		
36	m	68	0	0	x		70-80%			

Patient number	Sex	Age (y)	mRS (p)	NIHSS (p)	Stroke	TIA	GoS (%)			
							ICA right	ICA left	MCA right	MCA left
37	f	49	2	2	x		90%	90%		100%
38	f	58	0	0	x		70%	100%		
39	f	49	0	0	x		100%			
40	f	72	0	0	x					>80%
41	m	47	1	2	x		100%			
42	m	51	1	3	x			40%	100%	
43	m	51	1	3	x		100%			
44	m	39	3	7	x			100%		
45	f	41	0	0			50%	100%		
46	m	62	2	2	x		100%			
47	m	54	3	8	x		80%			
48	m	57	2	2	x		30-40%	30-40%	100%	
49	m	57	1	1	x			100%		
50	m	30	0	0		x			> 70%	
51	m	82	0	0	x			70-80%		
52	m	65	3	7	x		50%	100%		
53	m	57	0	0		x		100%		
54	m	68	0	0	x			100%		
55	f	42	0	1	x		50%	>70%		
56	f	74	0	0	x		30%	70%		
57	m	70	0	0	x		70%	50%		
58	m	53	0	0		x		100%		
59	m	74	0	0			80-90%	40%		
60	m	78	0	0	x		80-90%			
61	f	76	0	0	x			80%		

Patient number	Sex	Age (y)	mRS (p)	NIHSS (p)	Stroke	TIA	GoS (%)			
							ICA right	ICA left	MCA right	MCA left
62	m	67	0	0	x		<50%	70%		
63	f	43	0	0	x				80%	
64	f	54	0	0	x			100%		
65	m	61	0	0	x		50%	70-80%		
66	m	67	1	1	x		40%	100%		
67	m	74	2	2	x		70-80%	50%		
68	m	54	1	1	x			100%		

2 References

1. Martin SZ, Madai VI, Von Samson-Himmelstjerna FC, Mutke MA, Bauer M, Herzig CX, et al. 3D GRASE pulsed arterial spin labeling at multiple inflow times in patients with long arterial transit times: Comparison with dynamic susceptibility-weighted contrast-enhanced MRI at 3 Tesla. J Perinatol (2015) 392–401. doi:10.1038/jcbfm.2014.200
